# Propionic acid regulates immune tolerant properties in B Cells

**DOI:** 10.1111/jcmm.17287

**Published:** 2022-03-27

**Authors:** Gui‐Xiang Tian, Ke‐Ping Peng, Yong Yu, Cheng‐Bai Liang, Hai‐Qing Xie, Yu‐Yang Guo, Shan Zhou, Michael B. W. Zheng, Peng‐Yuan Zheng, Ping‐Chang Yang

**Affiliations:** ^1^ Department of Ultrasonic The Second Xiangya Hospital of Central South University Changsha China; ^2^ Research Center of Ultrasonography The Second Xiangya Hospital of Central South University Changsha China; ^3^ Department of Gastroenterology Fifth Affiliated Hospital Zhengzhou University Zhengzhou China; ^4^ 118393 Department of Otorhinolaryngology‐Head and Neck surgery The first Hospital Hunan University of Chinese Medicine Changsha China; ^5^ Department of Gastroenterology The Second Xiangya Hospital of Central South University Changsha China; ^6^ Department of Life Science McMaster University Hamilton Ontario Canada; ^7^ Guangdong Provincial Key Laboratory of Regional Immunity and Diseases Shenzhen China; ^8^ State Key Laboratory of Respiratory Disease Allergy Division at Shenzhen University Institute of Allergy & Immunology Shenzhen University School of Medicine Shenzhen China

**Keywords:** food allergy, immune regulation, intestine, propionic acid, short‐chain fatty acid

## Abstract

Interleukin 10 (IL‐10)‐producing B cells (B10 cells) are a canonical cell fraction for regulating other activities of immune cells. Posttranscriptional modification of IL‐10 in B10 cells is not yet fully understood. Short‐chain fatty acids play an important role to regulate the functions of immune cells. This study aims to clarify the role of propionic acid (PA), a short‐chain fatty acid, in regulating the expression of IL‐10 in B10 cells. Blood samples were collected from patients with food allergy (FA) and healthy subjects. Serum and cellular components were prepared with the samples, and analysed by enzyme‐linked immunosorbent assay and flow cytometry, respectively. The results showed that serum PA levels were lower in FA patients. PA concentrations were negatively correlated with serum cytokine Th2 concentrations, specific IgE concentrations in serum and skin prick test results. The peripheral frequency of B10 cells and the production of IL‐10 in B cells were also associated with serum PA concentrations. Activation of B cells by CpG induced the production of IL‐10 and tristetretrprolin (TTP), in which TTP caused the spontaneous decay of IL‐10 mRNA. PA was necessary to stabilize the IL‐10 mRNA in B cells by inducing the production of granzyme B, which resulted in the degradation of the IL‐10 mRNA. Administration of PA attenuated FA response in mice by maintaining homeostasis of B10 cells. In conclusion, PA is needed to stabilize the expression of IL‐10 in B10 cells. PA administration can mitigate experimental FA by maintaining B10 cell functions.

## 1 INTRODUCTION

Immune tolerance is a condition whereby the immune system does not respond to relevant stimulation as it should. The components of the immune tolerance system consist predominantly of immunotolerant cells and mediators. Immunotolerant cells include regulating T cells (Tregs) and regulating B cells (Bregs or B10 cells). Immune regulatory mediators (such as the transformation growth factor β or TGF‐β and IL‐10) are released by Tregs or/and B10 cells during proper activation. Disruption of immune tolerance is known to play a crucial role in immune diseases. Reduction in the number of immunotolerant cells and/or lower production of regulating cytokines play a crucial role in disease pathogenesis.^1^ But the factors responsible for the dysfunction of the immune regulatory system remain unclear.

The role of B10 cells in maintaining intestinal homeostasis has been recognized. By releasing IL‐10 during proper activation, B10 cells suppress other activities of immune cells to restrict immune response in an appropriate range. Published data show that B10 cell dysfunction is associated with the pathogenesis of food allergy (FA)^2^ and many other immune disorders.^3^ However, the factors responsible for B10 cell dysfunction are unclear; the remedies used to recover B10 cell functions are limited and require further investigation.

It is recognized that short‐chain fatty acids (SCFAs), such as acidic acid (AA), butyric acid (BA) and propionic acid (PA), plays an important part in maintaining homeostasis in the body.^4^ The intrinsic sources of SCFAs are mainly from intestinal bacterial metabolites. SCFA have immune regulatory functions, such as inhibiting histone acetylase (HDAC), activating G‐coupled receptors and serving as energetic substrates.^5^ BA is a pan histone deacetylase inhibitor, and protects against cancer and inflammation.^6^ AA and PA also have inhibitory effects on HDACs and involve in immune regulation.^5^ It is reported that the PA administration effectively prolongs full‐thickness skin grafts.^7^ GRP40 to GRP43 are the receptors of SCFAs.^5^

Interleukin‐10 derived from immunoregulatory cells is known to play an essential role in immune regulation. However, IL‐10 therapies were not used in the clinic yet.^8^ Our previous work showed that the spontaneous IL‐10 mRNA decay in B10 cells.^9^ Other investigators also pointed to this phenomenon.^10^, ^11^ Insufficient supply of SCFA is associated with the pathogenesis of immune disorders.^12^ We hypothesize that the short of SCFAs may involve in the instability of IL‐10 in B10 cells. Thus, we analysed blood samples from patients with food allergy (FA). The results showed that lower serum levels of propionic acid (PA), one of the SCFAs, were negatively associated with the frequency of peripheral B10 cells. The PA supplement effectively inhibited the spontaneous decay of IL‐10 mRNA in B cells, and suppressed experimental FA.

## 2 MATERIALS AND METHODS

### 2.1 Reagents

Reagent kits of GPR41 and GPR43 shrine, antibodies (Abs) of TTP (clone#: A‐8), ubiquitin (P4D1), IL‐10 (3C12C12, AF540), granzyme B (2C5), CD19 (F‐3, AF488), CD5 (UCH‐T2, AF594), CD45 (3Z‐45, AF647) were purchased from Santa Cruz Biotech. ELISA kits of ovalbumin‐specific IgE, IL‐4, IL‐5, IL‐10, IL‐13, IFN‐γ, EPO and MCP‐1 were purchased from BioMart. CpG ODN 1826 (5′‐TCCATGACGTTCCTGACGTT‐3′), reagents and materials for RT‐qPCR, immunoprecipitation and Western blotting were purchased from Invitrogen. Indole‐3‐propionic acid (soluble in ethanol at 50 mg/ml) was purchased from Sigma Aldrich. AS101 was purchased from Wyeth‐Ayerst Research (Radnor).

### 2.2 Human subjects

Patients with FA were recruited at the First Affiliated Hospital of Shenzhen University (Shenzhen, China) and the Fifth Affiliated Hospital of Zhengzhou University (Zhengzhou, China) from March 2020 to March 2021). Diagnosis and management of FA have been performed by our physicians following routine procedures, including history of FA, positive skin pitting (SPT) and serum‐specific positive IgE. Patients with any of the following conditions were excluded, including using immune suppressors for any reasons, autoimmune diseases, severe organ diseases and cancers. Age and gender‐matching healthy control (HC) subjects were also recruited. The experimental procedures were approved by the Human Ethical Committee at Shenzhen University and Zhengzhou University. Written informed consent was received from every human subject. The demographic data are presented in Table 1.

**TABLE 1:** Demographic data of human subjects

Items	FA	HC
Male/female	20/20	20/20
Age: Median (IQR)	33.3 (22.2, 44.8)	31.2 (23.5, 44.4)
FA history (subjects)	40	0
SPT positive	40	0
Serum IgE (IU/ml)	365.8 ± 113.6	355.3 ± 113.6
Specific IgE positive	40	0
Immunotherapy history	0	0
Steroid therapy history	0	0
Co‐suffer allergy		
Allergic asthma	1	0
Allergic dermatitis	1	0
Allergic rhinitis	2	0
Blood eosinophil (10^9^/L)	0.36 (0.21, 0.54)	0.11 (0.05, 0.14)*
Blood neutrophil (10^9^/L)	5.46 (4.91, 6.88)	5.65 (4.81, 6.26)

Note

Data are presented as mean ± SEM or median (IQR). *p < 0.001, compared with FA patients.

### 2.3 SPT

Skin pitting was performed for all human subjects. Food allergens (Cow's milk, egg white, egg yolk, wheat flour, soybean, carrot, potato and peanut) were purchased from Allergopharma (Germany) and used in SPT. Saline and histamine (10 mg/ml) were used as negative and positive controls in SPT, respectively. The results of SPT were observed and recorded 15 min later. The SPT‐positive criterion was set if the mean diameter of wheal was ≥3 mm larger than the negative control. The largest wheal size was recorded for each patient. The SPT results are presented in Table S1.

### 2.4 Assessment of serum‐specific IgE

The serum‐specific IgE (sIgE) levels were determined by ImmunoCap (for human; Allergopharma) or ELISA (for mice) with commercial reagent kits following the manufacturer's instructions. The positive sIgE criterion for human samples was 0.35 kU/L.

### 2.5 ELISA (Enzyme‐linked immunosorbent assay)

Cytokine levels in the serum or supernatant were determined by ELISA with commercial reagent kits following the manufacturer's instructions.

### 2.6 Preparation of peripheral blood mononuclear cells (PBMCs)

Blood samples were collected from each human subject through the ulnar vein puncture. PBMCs were isolated from blood samples by Percoll gradient density centrifugation. Serum samples were collected and stored at −80°C until use.

### 2.7 Cell culture

Cells were cultured in RPMI1640 medium supplemented with 10% foetal calf serum, 0.1 mg/ml streptomycin, 100 U/ml penicillin, and 2 mM glutamine. Cell viability was 98%–100% as assessed by the Trypan blue exclusion assay.

### 2.8 Flow cytometry (FACS)

In the surface staining, cells were stained with fluorescence labelled antibodies (diluted to 1 ug/ml) or isotype IgG for 30 min at 4°C. Cells were washed with phosphate‐buffered saline (PBS) 3 times, and analysed with a FACS device (BD FACSCanto II). In the intracellular staining, cells were fixed with 1% paraformaldehyde (containing 0.05% Triton x‐100) for 1 h, washed with PBS 3 times. The cells were then processed with the same procedures of surface staining. The data were analysed with software package Flowjo (TreeStar Inc.). The data obtained from isotype IgG staining were used as gating references.

### 2.9 Isolation of B cells

Preparation of peripheral blood mononuclear cells were labelled with antibodies of CD45 (AF647), CD19 (AF488) and CD5 (AF594). In FACS, CD45^+^ cells were gated first; followed by gating and harvesting CD19^+^ cells (used as B cells), or CD19^+^ CD5^+^ cells (used as B10 cells). The post‐FACS check results showed that the purity of isolated B cells and B10 cells was 96%–98%.

### 2.10 FA mouse model development

BALB/c mice (6–8‐week‐old) were purchased from the Guangzhou Experimental Animal Center, and maintained in a specific pathogen‐free facility. The mice were allowed to access food and water freely. The animal experimental procedures were approved by the Animal Ethical Committee at Shenzhen University.

To develop the FA model, mice were sensitized by subcutaneous injection with ovalbumin (OVA, 100 µg/mouse in 0.1 ml Alum) on the back skin on day 0 and day 3, respectively. The immunization was boosted by gavage‐feeding mice with OVA (1 mg/mouse in 0.3 ml saline) daily from day 9 to day 13. Mice were oral‐challenged with OVA (5 mg/mouse in 0.3 ml saline) on day 15 (or started the treatment with PA). Core temperature was recorded 30 min after the challenge with a rectal thermometer. Diarrhoea was recorded in the period of 2 h after the challenge. The truncated blood was collected. The serum was separated from blood samples, and stored at −80°C until use. After the sacrifice, a 15‐cm jejunum segment was excised, and rinsed with 3 ml saline in a syringe; the fluid was recovered, centrifuged at 10,000 *g* for 10 min at 4°C; supernatant was collected, and used as gut lavage fluid (GLF). The jejunal segments were then cut into small pieces, incubated with collagenase IV (1 mg/ml) at 37°C for 30 min with mild agitation. Single cells were collected by filtering through a cell strainer (100 µm first, then 40 µm), and used for further experiments.

### 2.11 Treating FA mice with Indole‐3‐Propionic Acid (PA, in short)

Two days after the last OVA challenge (day 15), FA mice received daily intraperitoneal injections of either PA (30 mg/kg) or saline (control) or AS101 (an IL‐10 inhibitor; 10 µg/mouse in 0.1 ml saline, ip) for 7 days.

### 2.12 Assessment of SCFAs

Following published procedures,^13^ the serum levels of SCFAs were analysed by high‐performance liquid chromatography (HPLC; Merck Hitachi) with a Rezex ROA‐Organic Acid H^+^ ion exchange column together with a SecurityGuard Cartridges Carbo‐H from Phenomenex; the flow rate was 0.4 ml at 40°C; the eluent solution was H_2_SO_4_ (10 mmol/L). The samples were quantified against the SCFA standards (purchased from Sigma Aldrich).

### 2.13 Immunoprecipitation (IP)

Proteins were extracted from cells collected from relevant experiments, and precleared by incubating with protein G agarose beads for 2 h, followed by centrifugation at 10,000 *g* for 10 min to remove the beads. Supernatant was collected and incubated with an anti‐TTP Ab (1 µg/ml) overnight. Immune complexes were precipitated by incubating with protein G agarose beads for 2 h. The beads were collected by centrifugation at 10,000 *g* for 10 min. Proteins on the beads were eluted with an eluting buffer, and analysed by Western blotting. All the IP procedures were performed at 4°C.

### 2.14 Mass spectrometry (MS)

Protein samples precipitated by anti‐TTP Ab were sent to the MS centre at Shenzhen University to be analysed by MS. The analysing procedures were carried out, and the MS data were analysed by professional staff at the MS centre. Briefly, the samples were loaded on C18 nanoLC trap column (100μm × 3cm, C18, 3μm, 150 Å) and washed by Nano‐RPLC Buffer A (0.1%FA, 2%ACN) at 2 μl/min for 10 min. Data acquisition was performed with a Triple TOF 5600 System (AB SCIEX). Based on combined MS and MS/MS spectra, proteins were successfully identified based on 95% or higher confidence interval of their scores in the MASCOT V2.3 search engine (Matrix Science Ltd.), using the following search parameters: Cow‐ lacbobacillus casel mix database.

### 2.15 Real‐time quantitative RT‐PCR (RT‐qPCR)

Total RNA was isolated from cells collected from relevant experiments or from IP products with the TRIzol reagents. Complementary DNA (cDNA) was synthesized with a reverse transcription kit following the manufacturer's instruction, which was served as a template for RT‐qPCR using SYBR Green Master Mix. Primers for real‐time PCR were synthesized by Shanghai Sangon Biotech, including granzyme B (tgacagtgcaggaagatcga and ataggagacaatgccctggg), TTP (gactgagctatgtcggacct and ggttgtggatgaagtggcag), and IL‐10 (gccaagccttgtctgagatg and aagaaatcgatgacagcgcc). The amount of the target gene mRNA was calculated by the method of 2^−∆∆Ct^ and normalized against the housekeeping gene β‐actin mRNA.

### 2.16 Western blotting

Proteins were prepared from cells collected from relevant experiments, separated by SDS‐PAGE (sodium dodecyl sulphate – Polyacrylamide gel electrophoresis), and transferred onto a PVDF membrane. After blocking with 5% skim milk for 30 min, the membrane was incubated with primary antibodies of interest (detailed in figures) diluted to 200 ng/ml overnight at 4°C, washed with TBST (Tris‐buffered saline containing 0.05% Tween 20) 3 times, incubated with horseradish peroxidase‐labelled antibodies (20 ng/ml) for 2 h at room temperature, washed with TBST 3 times. Immunoblots on the membrane were developed with the enhanced chemiluminescence and photographed in an imaging station (UVP).

### 2.17 RNA interference (RNAi)

The expression of GPR41 and GPR43 in B cells was knocked down by RNAi with commercial shrine reagent kits following the manufacturer's instructions. Effects of RNAi were checked 48 h after the transfection by Western blotting.

### 2.18 Statistics

The data are presented as mean ± SEM or median (IQR). The difference between two groups was determined by Student *t*‐test or ANOVA followed by Dunnett's test or Bonferroni test. The Spearman correlation coefficient test was performed to determine correlation between two group data. *p *< 0.05 was set as the significant criterion.

## 3 RESULTS

### 3.1 Serum PA levels are negatively correlated with serum Th2 cytokine levels in AF patients

**FIGURE 1:**
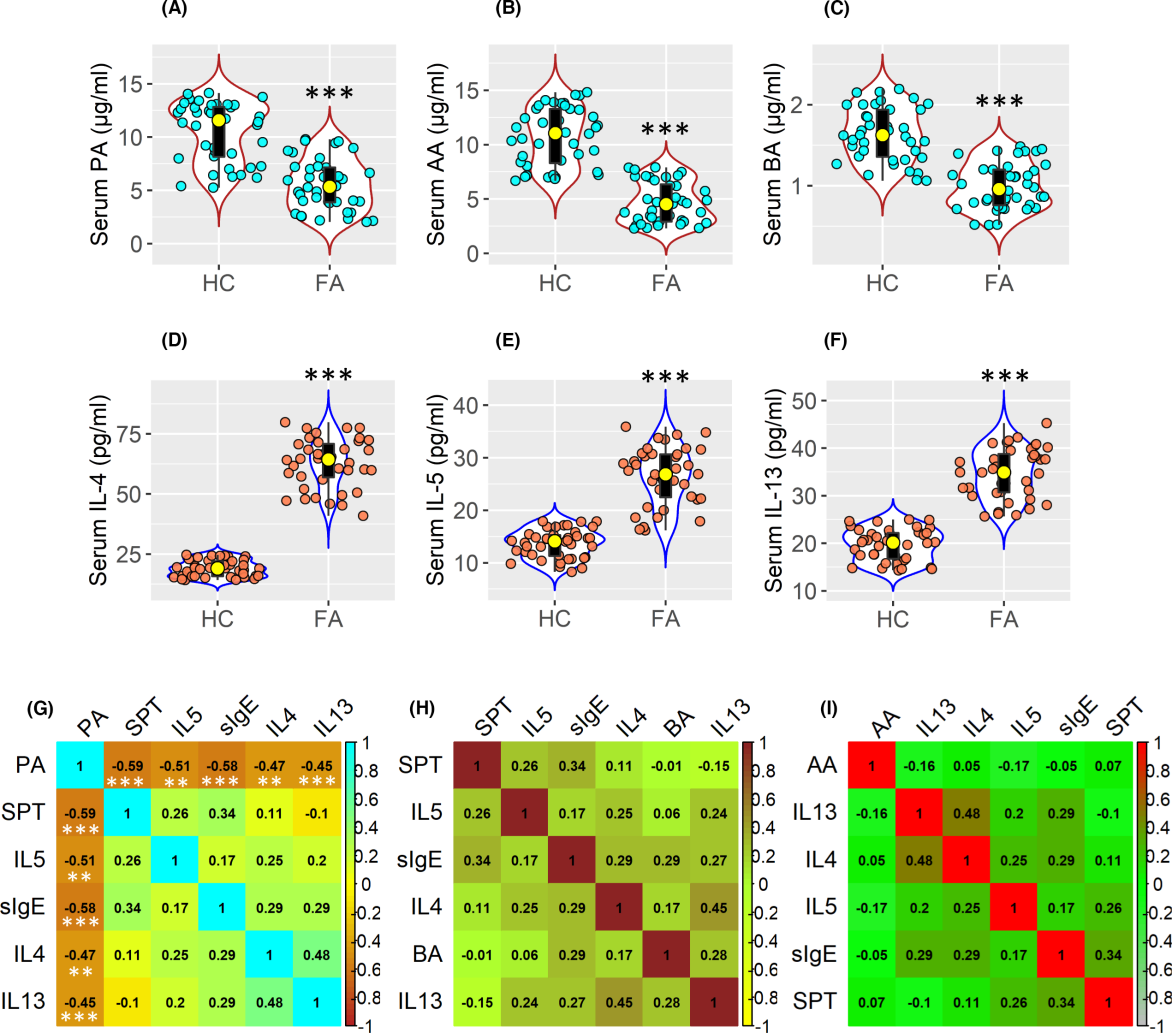
Association between serum PA levels and Th2 response. Serum samples obtained from 40 FA patients and 40 HC (healthy control) subjects were analysed by ELISA. (A–C) Serum levels of propionic acid (PA), acetic acid (AA) and butyric acid (BA). (D–F) Serum levels of Th2 cytokines. (G–I) Heatmap show correlation between serum PA (G), or BA (H), or AA (I) versus IL‐4, IL‐5, IL‐13, SPT wheal size, and serum sIgE (the original data are presented in panels A–F). The numbers in heatmaps indicate correlation coefficient. The original data of wheal size and sIgE are presented in Table S2 and Table S3 The data of violin plots and boxplots are presented as median (IQR). Each bubble in violin plots and boxplots presents data obtained from one sample. ***p < 0.001 (Mann–Whitney test), compared with the HC group (A–F). In panel G–I, **p < 0.01, ***p < 0.001; Spearman correlation coefficient test

Blood samples were collected from 40 FA patients and 40 healthy controls (HC) subjects. The serum was isolated from the samples and was analysed by HPLC and ELISA. In comparison with HC samples, serum butyric acid (BA), acetic acid (AA) and propionic acid (PA) concentrations were lower in FA samples than in HC samples (Figure 1A–C). Cytokine analysis showed greater Th2 cytokine levels, a dominant Th2 profile, in FA samples than in HC samples (Figure 1D–F). A negative correlation was found between serum Th2 cytokine levels, specific IgE (sIgE) levels, SPT size and the PA levels (Figure 1G), but not either AA or BA (Figure 1H–I). The results implicate a link between the lower serum PA levels and the Th2 polarization in FA patients.

### 3.2 Serum PA concentrations are associated with the peripheral frequency of B10 cells and the production of IL‐10

**FIGURE 2:**
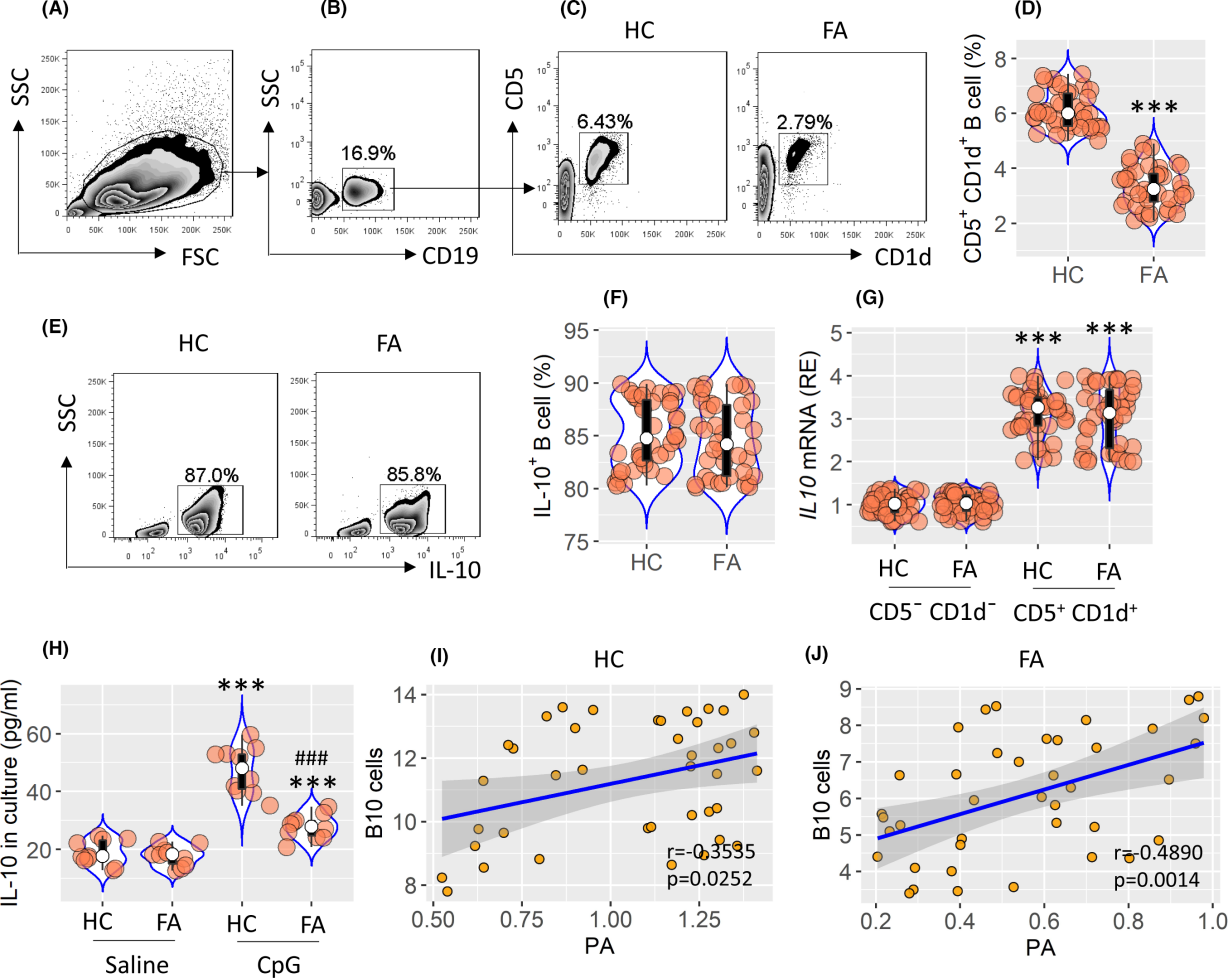
Lower serum PA levels are associated with lower B10 cell frequency in FA patients. Peripheral blood mononuclear cells (PBMCs) were obtained from 40 FA patients and 40 HC (healthy control) subjects, and analysed by FACS. (A) The SSC/FSC plots. (B) Gated plots show CD19^+^ B cell frequency. (C) Gated plots show CD5^+^ CD1d^+^ B cell frequency. (D) Violin plots show median (IQR) of CD5^+^ CD1d^+^ B cells from 40 HC subjects and 40 FA subjects. (E and F) FACS plots show IL‐10^+^ B cells in CD19^+^ CD5^+^ CD1d^+^ B cells (as shown in panel C). Violin plots show summarized B10 cell counts. (G) CD19^+^ CD5^+^ CD1d^+^ B cells were isolated from PBMCs, and analysed by RT‐qPCR. Violin plots show IL‐10 mRNA levels in B10 cells. (H) B10 cells were isolated from 10 HC subjects and 10 FA subjects, and cultured in the presence of CpG (1 µg/ml) or saline overnight. Violin plots show median (IQR) of IL‐10 levels in culture supernatant from 10 tests. (I–J) Positive correlation between serum PA levels and peripheral B10 cell frequency in the HC group and the FA group. Each bubble in violin plots presents data obtained from one sample. ***p < 0.001, compared with the HC group (D), or the CD5¯ CD1d¯ B cell group (G), or the saline group (H). ^###^ *p *< 0.001, compared with the HC/CpG group

With CD1d^+^ CD5^+^ as B10 cell markers,^2^ the frequency of B10 cells was found to be lower in the FA group than in the HC group (Figure 2A–D). Over 80% of CD1d^+^ CD5^+^ B cells also expressed IL‐10, which can be called B10 cells (Figure 2E,F). While B10 cells of HC subjects and FA patients expressed IL10 mRNA almost equally (Figure 2G), the production of IL‐10 was lower in the B10 cells sampled from the FA group (Figure 2H). Positive correlation was detected between serum PA levels and the peripheral frequency of B10 cells (Figure 2I,J). The findings suggest that serum PA may be associated with homeostasis in B10 peripheral cells.

### 3.3 PA regulates the expression of IL‐10 in B10 cells

**FIGURE 3:**
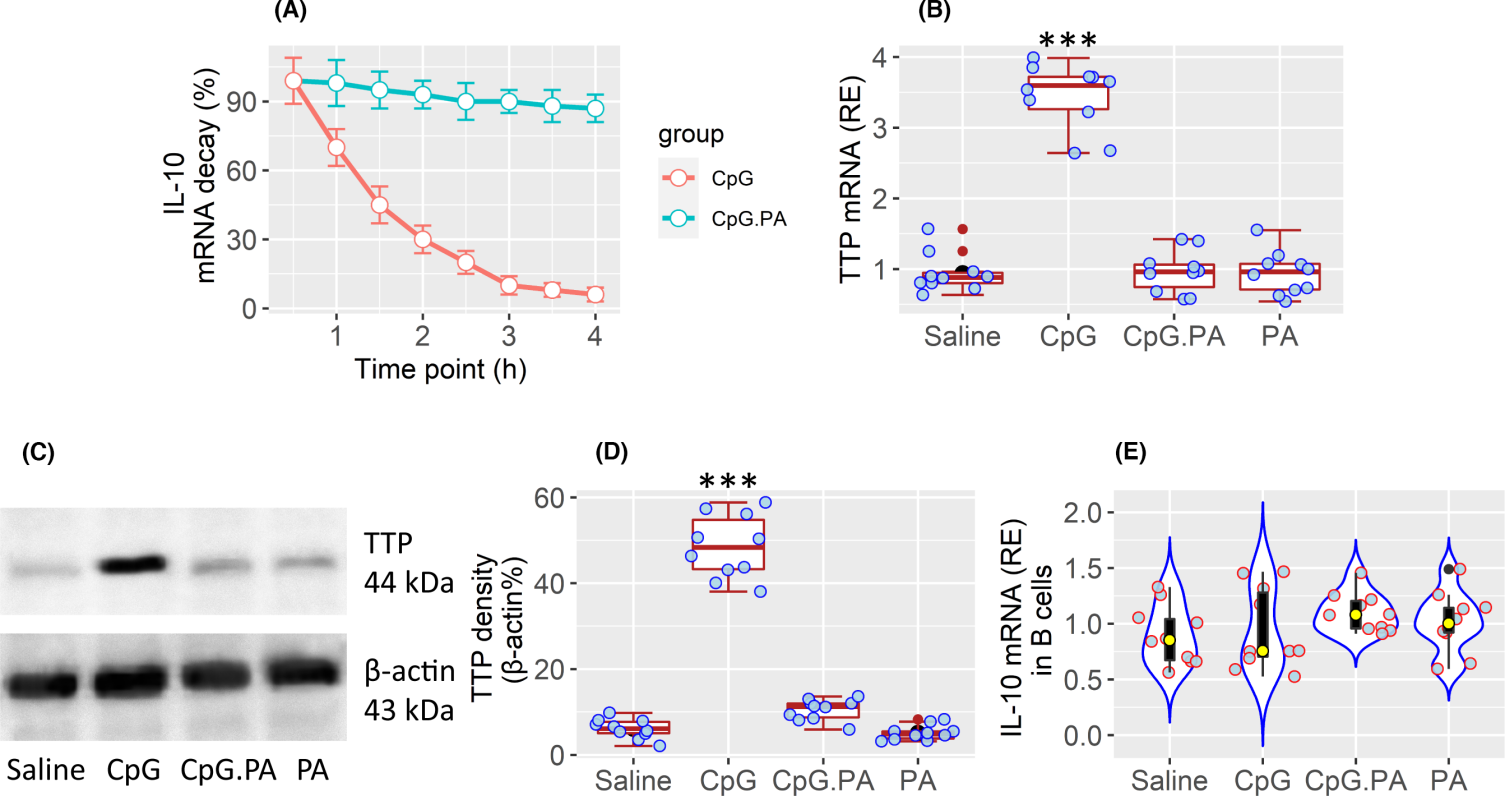
Propionic acid blocks spontaneous IL‐10 mRNA decay in B cells. CD19^+^ CD25¯ B cells were isolated from blood samples collected from HC subjects. (A) IL‐10 mRNA levels in B cells collected at different time points after exposure to CpG (used as an IL‐10 inducer; 1 µg/ml) or both CpG and PA (10 µM). (B–D) 4 h after culturing with fresh medium, B cells were collected and analysed by RT‐qPCR and Western blotting. TTP mRNA levels (B), protein levels (C) and TTP blot integrated density (D) in B cells after the treatment denoted on the x axis overnight. (E) Violin plots show IL‐10 mRNA levels bound by TTP protein (analysed from IP products by RT‐qPCR with equal protein amounts per group). No statistical difference between groups of panel E. The data of B, D and E are presented as median (IQR). Each bubble in boxplots and violin plots presents data obtained from one sample. ***p < 0.001 (ANOVA + Dunnett's test), compared with the saline group

The data of Figure 1 and Figure 2 suggest that the PA may involve in the regulation of IL‐10 expression in B10 cells. To test this, B cells were isolated from blood samples of 10 HC subjects, exposed to CpG (an IL‐10 inducer^14^) in culture for 3 h to induce the IL‐10 expression. The cells were then washed, cultured in fresh medium, with or without the presence of PA. The B cells were collected at various times and processed for RT‐qPCR analysis. We found that exposure of B cells to CpG 30 min later, the IL‐10 mRNA levels in B cells began to decline. The reduction of IL‐10 mRNA was progressively decreased below 10% of the baseline levels within 4 h. The results indicate that IL‐10 mRNA decay occurs spontaneously in B cells. The presence of PA prevented the spontaneous decline of IL‐10 mRNA in B cells (Figure 3A). Tristetraproline (TTP) has been known to destabilize IL‐10.^9^, ^11^ We found that the TTP levels in B cells were increased upon exposing to CpG; such an effect was counteracted by the presence of PA. Exposure to PA alone did not alter the TTP levels in B cells (Figure 3B–D). The results suggest that, in line with previous reports,^9^, ^11^ TTP is also responsible for the IL‐10 mRNA decay in this experimental setting, and PA can counteract with the effects of TTP. Additionally, by immunoprecipitation assay with anti‐TTP antibody as a bait, we observed that TTP protein (extracted from the B cells) bound IL‐10 mRNA (Figure 3E).

### 3.4 GPR43 mediates the effects of PA on stabilizing IL‐10 mRNA in B cells

**FIGURE 4:**
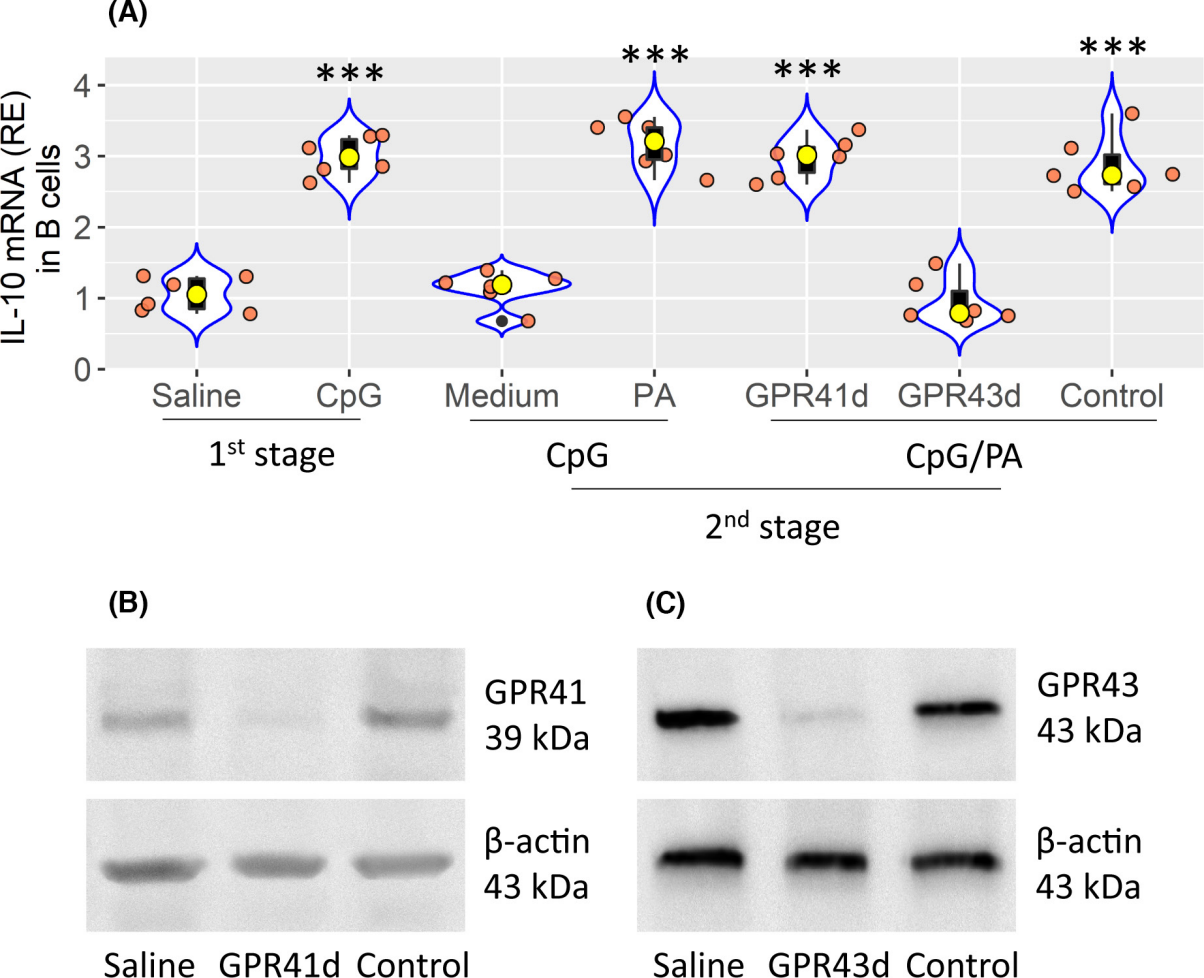
GPR43 mediates effects of PA on stabilizing IL‐10 mRNA in B cells. CD19^+^ CD25¯ B cells were isolated from blood samples collected from HC subjects. (A) The B cells were cultured in the treatment denoted on the x axis. 1st stage: B cells were cultured in the presence of CpG (1 µg/ml) overnight. 2nd stage: After culturing with CpG overnight, B cells were washed with medium, and cultured with fresh medium for 4 more h. B cells harvested at both stages were analysed by RT‐qPCR to determine the IL‐10 mRNA levels. Violin plots show IL‐10 mRNA levels in B cells. PA: 10 µM. GPR41d (GPR43d): GPR41‐ or GPR43‐deficient B cells (prepared by RNAi). Control: B cells were treated with control RNAi reagents. ***p < 0.001 (ANOVA + Dunnestt's test), compared with the saline group. The data of violin plots are presented as median (IQR). Each bubble in violin plots presents data obtained from one sample. (B and C) Immunoblots show RNAi results of GPR41 (B) and GPR43 (C)

GPR41 and GPR43 are the main receptors of PA.^15^ To elucidate which of them involves in stabilizing IL‐10 mRNA, we knocked down the GPR41 or GPR43 expression in B cells by RNAi, then exposed the GPR41 knockdown or GPR43 knockdown B cells to CpG and PA. The results showed that the IL‐10 mRNA decay was observed in GPR41^+^ GPR43⁻ B cells, but not in GPR43^+^ GPR41¯ B cells (Figure 4). The results indicate that GPR43 mediates the PA effects on stabilizing IL‐10 mRNA in B cells.

### 3.5 PA induces granzyme B (GrB) expression to inhibit TTP in B cells

**FIGURE 5:**
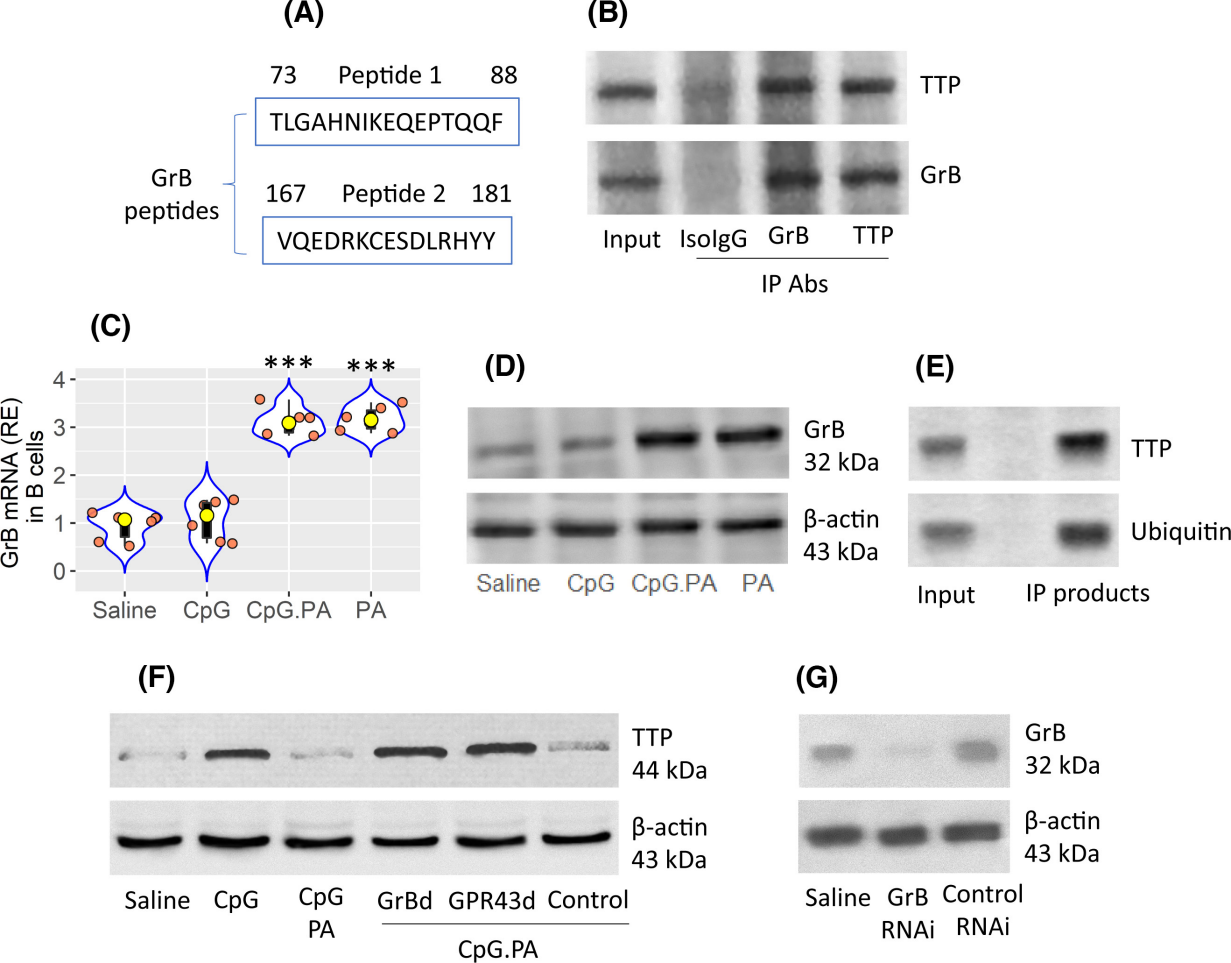
Propionic acid induces GrB to suppress TTP in B cells. (A) MS results show GrB peptides in B cell‐derived IP products precipitated by a TTP Ab. (B) IP results show a complex of GrB/TTP in CpG‐primed B cells. IsoIgG: Isotype IgG (a control Ab). (C and D) GrB mRNA (C) and GrB protein (D) in B cells after the treatments denoted on the x axis of panel C. (E) Colocalization of TTP/ubiquitin in B cells primed by CpG/PA. (F) TTP protein levels in B cells after the treatments denoted below the gels. (G) Results of GrB RNAi in B cells. The data of violin plots are presented as median (IQR). ***p < 0.001 (ANOVA + Dunnett's test), compared with the saline group. Each bubble in violin plots presents data obtained from one sample. All the experiments were repeated 3 times

To better understand the mechanism by which TTP regulates the stability of IL‐10 mRNA in B cells, B cells were treated with CpG and PA in culture. Protein extracts were prepared with the B cells, and immunoprecipitated (IP) with an anti‐TTP antibody as a bait. The IP products were analysed by mass spectrometry (MS). MS results showed that it was GrB (Figure S1) to form a complex with TTP in B cells (Figure 5A). Guided by the MS results, the TTP/GrB complex in the IP products was verified by Western blotting (Figure 5B). We then analysed RNA and protein extracts of B cell by RT‐qPCR and Western blotting. The results showed that after exposure to PA in the culture, the expression of GrB was increased in B cells, which did not occur in those exposed to CpG alone (Figure 5C,D). Because GrB is a protease, the results suggest that the binding of GrB and TTP can lead to degradation of TTP. We indeed colocalized TTP and ubiquitin in the complex of GrB (Figure 5E). Treating GrB‐deficient or GPR43‐deficient B cells with PA did not suppress the CpG‐increased TTP (Figure 5F–G). The results demonstrate that PA induces GrB expression to suppress TTP in B cells, and thus, to contribute to IL‐10 mRNA stabilization (Figure S2).

### 3.6 Administration of PA attenuates FA in mice by maintaining the homeostasis of B10 cells

**FIGURE 6:**
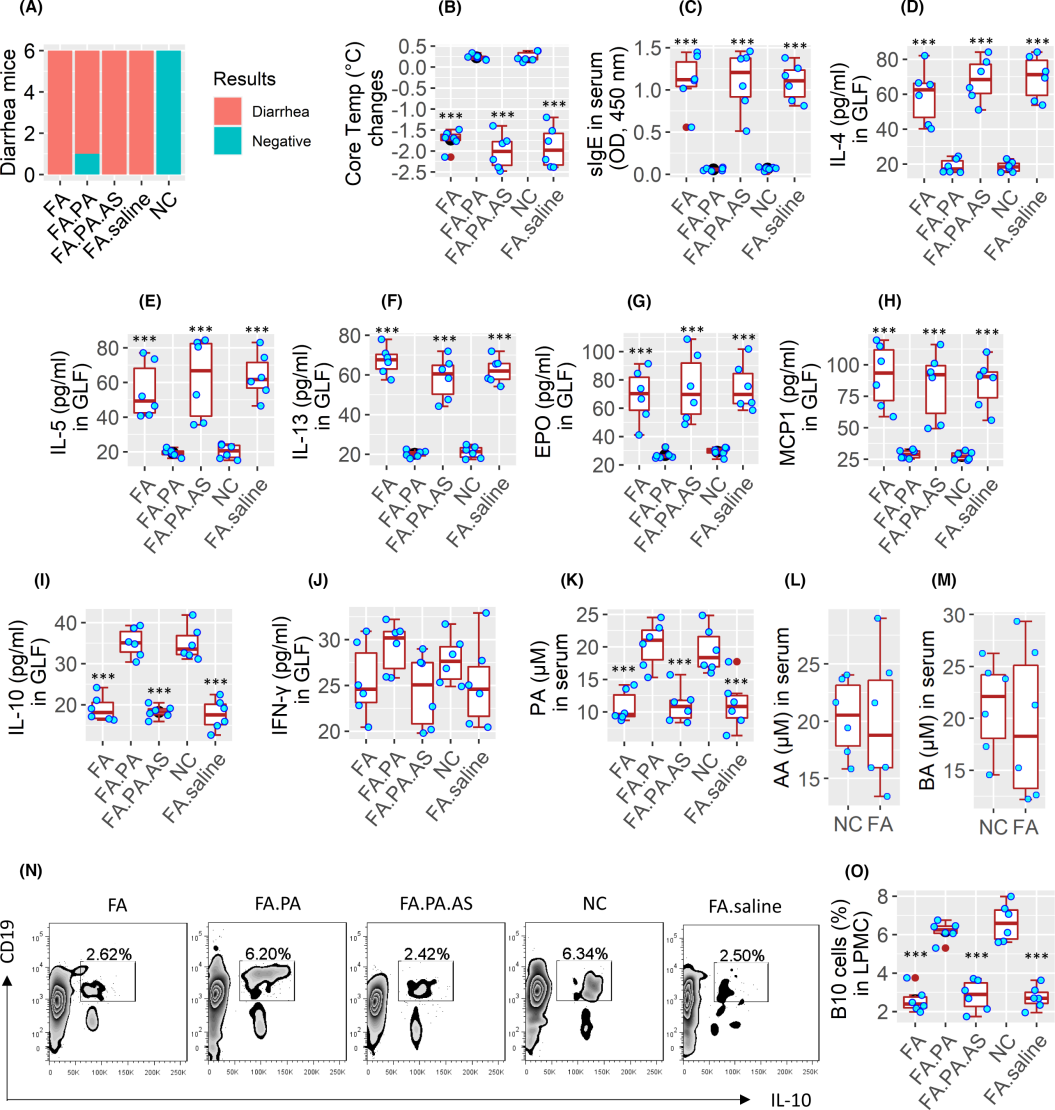
Assessment of the role of PA in alleviating experimental FA. One day after the last oral challenge, FA mice (6 mice per group) were treated with PA (indole‐3‐PA) or PA and AS (AS101, an IL‐10 inhibitor), or saline (control) for one week. (A) Diarrhoea mice (recorded in a 2‐h period after the last antigen challenge). (B) Core temperature changes at 30 min after the last antigen challenge. (C) Serum‐specific IgE levels. (D–I) Gut lavage fluid (GLF) levels of Th2 cytokines (D–F), eosinophil peroxidase (EPO, G), mast cell protease‐1 (MCP1, H), IL‐10 (I) and IFN‐γ (J). (K) Serum PA levels. (L–M) serum AA and BA levels. (N) Gated FACS plots show B10 cell frequency in LPMCs. (O) Median (IQR) of B10 cell counts in LPMCs of 6 mice per group. ***p < 0.001 (ANOVA + Dunnett's test), compared with the NC group. Each bubble in boxplots presents data obtained from one mouse

Then, we did an in vivo study to look at the role of PA in maintaining homeostasis in B10 cells. An FA mouse model was developed (Figure S3). Upon challenging with a specific antigen, FA mice showed the FA response, including diarrhoea (Figure 6A), core temperature drop (Figure 6B), high serum‐specific IgE levels (Figure 6C), Th2 polarization and high levels of allergic mediators in the gut lavage fluid (GLF) (Figure 6D–H). IL‐10 levels were lower and IFN‐γ levels were not altered (Figure 6I,J). FA mice also showed lower serum PA levels, but not AA or BA levels (Figure 6K–M), lower frequency of B10 cells in the intestinal tissues (Figure 6N,O). Administration of PA (Indole‐3‐PA) for one week markedly attenuated the FA response, increased B10 cells in the intestine and increased GLF IL‐10 levels. The therapeutic effects of PA were abolished by inhibiting IL‐10 with AS101 (Figure 6). The results show that PA plays an important role in the maintenance of B10 cell homeostasis in the intestine.

## 4 DISCUSSION

This study revealed that PA, one of the SCFAs, played an important role in the stabilization of IL‐10 posttranscriptional modification in B cells. We found the serum PA levels were lower in FA patients, which were negatively correlated with the Th2 polarization, sIgE levels in the serum and the SPT results. Activation of B cells by CpG induced the production of both IL‐10 and TTP, the latter caused IL‐10 mRNA spontaneous decay, which could be abrogated by the presence of PA. The underlying mechanism was that PA induced granzyme B (GrB) expression in B cells; GrB formed a complex with TTP to induce TTP degradation, thereby stabilizing IL‐10 expression.

Lower serum levels of SCFA, including AA, BA and PA, were found in patients with FA. This is in line with previous investigations. Such as Sandin et al reported that lower faecal AA, BA and PA levels were associated with the pathogenesis of FA in human early life.^16^ Roduit et al reported that the faecal SCFA levels in children at 1‐year age were significantly negatively associated with suffering allergic diseases between 3‐ and 6‐year‐olds.^17^ This may be because the allergic environment affects the microbiota constitutions in the intestine, that reduces the SCFA‐producing bacteria in the intestine^18^ as SCFAs are produced by bacteria in the intestine, that are absorbed into the blood stream by colon epithelial cells. Our data show that the lower SCFS levels in the serum also occur in adult life, of which PA is significantly negatively correlated with serum Th2 cytokine levels, serum sIgE and SPT results. Current data show that the serum levels of PA, AA and BA are lower in FA patients, FA mice show lower serum PA levels, but the AA or BA levels are not altered; whether these AA and BA regulate other immune cell functions is warranted to further investigated.

The data show the lower frequency of B10 cells in FA patients. We found more than 80% CD1d^+^ CD5^+^ B cells expressed IL‐10. This is in line with previous studies. Noh et al reported that the frequency of peripheral B10 cells (also designated Br1 cells) was lower in FA patients.^2^ Our previous work demonstrated that the frequency of FA mouse B10 cells was lower in intestinal tissues.^9^ It is the consensus that B10 cells are one of the canonical immune regulatory cell fractions.^1^ By releasing the immune regulatory cytokine IL‐10, B10 cells suppress other immune cell activities to restrict immune responses within a p roper range.^1^ Failure to suppress abnormal immune responses can lead to immune diseases. The present data also show that the serum Th2 cytokine levels are significantly higher in FA patients, which are negatively correlated with peripheral B10 cell frequency. This implies a link between the low frequency of B10 cells and the state of Th2 polarization in FA patients.

We found that serum PA levels, but not AA or BA, are negatively correlated with peripheral B10 cell frequency. This suggests that the lower serum PA levels may be associated with the lower B10 cell frequency in FA patients. By reviewing the literature, this phenomenon has not been reported. The expression of IL‐10 is the cornerstone of B10 cell development and B10 cell functions. Current data show that the role of AP in regulating the expression of IL‐10 in B cells. The data have expanded our previous findings,^9^ in which we found that TTP caused the spontaneous IL‐10 mRNA decay in B cells. Based on the prevention of TTP‐induced IL‐10 mRNA decay in B cells, we propose that PA contributes to the stabilization of IL‐10 expression in B cells. It has been recognized that many factors can induce IL‐10 expression in B cells, such as LPS, one of the common microbial products.^19^ Therefore, it should be easy to induce B10 cells as LPS distributes to the human living environment extensively. However, the Th2 polarization status in FA patients as shown by the present data suggests that, at least partially, the lower serum levels of PA are associated with the B10 cell system dysfunction, and that consequently, induce Th2 polarization and FA.

Both GPR41 and GPR43 can be recognized by the PA, AA and BA.^20^ GPR43 is not only expressed by epithelial cells, but also expressed by immune cells.^21^ This data show that GPR43, but not GPR41, is responsible for mediating the role of PA in stabilizing IL‐10 stabilization in B cells. The underlying mechanism is that PA ligates GPR43 to induce GrB expression in B cells, of which the signal transduction pathway still needs to be further investigated. GrB forms a complex with TTP to induce TTP degradation, and thus, contribute to the IL‐10 stabilization.

With an FA mouse model, we verified the role of PA in stabilizing the IL‐10 expression in B cells and the benefit in alleviating FA. Although the immunopathological features of the FA and other allergic diseases have been well documented, treatments are still unsatisfactory. The efficiency of specific allergen immunotherapy still needs to be improved. Previous studies indicate that administration of Indole‐3‐PA can suppress inflammation or aberrant cell activities, such as attenuating steatohepotitis,^22^ protecting against radiation toxicity,^23^ reducing weight gain^24^ and modulating mitochondrial functions.^25^ This data indicate that administration of Indole‐3‐PA efficiently alleviates experimental FA. This suggests that administration of Indole‐3‐PA or having PA‐producing diet can be employed in the treatment of allergic diseases.

There are many phenotypes of B cells have been recognized. Such as CD24^high^ CD38^high^ B cells, CD24^high^ CD27^+^ B cells, CD27^int^ CD38^high^ B cells and CD38^+^ CD1d^+^ IgM^+^ CD147^+^ GZMB^+^ B cells. These B cell subsets have various immune regulatory activities.^3^ Whether PA also regulate the properties of these B cell subsets is of interest, and can be investigated in the future.

In summary, current data show that serum PA levels are negatively associated with Th2 polarization. PA induces GrB expression in B cells, which suppresses TTP and stabilizes IL‐10 expression. Administration of Indole‐3‐PA alleviates experimental FA.

## CONFLICT OF INTEREST

None to declare.

## AUTHOR CONTRIBUTIONS

**Gui Xiang Tian:** Conceptualization (equal); Data curation (equal); Funding acquisition (equal); Resources (equal); Writing – original draft (lead); Writing – review & editing (equal). **Ke‐Ping Peng:** Data curation (equal); Software (equal); Writing – review & editing (equal). **Yong Yu:** Data curation (equal); Resources (equal). **Cheng‐Bai Liang:** Data curation (equal); Resources (equal). **Hai‐Qing Xie:** Data curation (equal); Resources (equal); Software (equal). **Yu‐Yang Guo:** Data curation (equal); Software (equal). **Shan Zhou:** Data curation (equal); Software (equal). **Michael BW Zheng:** Conceptualization (equal); Data curation (equal); Formal analysis (equal); Resources (equal); Writing – review & editing (equal). **Peng‐Yuan Zheng:** Conceptualization (equal); Writing – review & editing (equal). **Ping‐Chang Yang:** Conceptualization (equal); Funding acquisition (equal); Supervision (equal); Writing – review & editing (equal).

## Supporting information

Supplementary Material

## Data Availability

All the data are included in this paper and the online supplementary materials.
