# Supplementary material for: Propionic acid regulates immune tolerant properties in B Cells
Source: J Cell Mol Med. 2022 Mar 27;26(10):2766–76. doi: 10.1111/jcmm.17287 (PMC9097846; doi:10.1111/jcmm.17287)
Supplement: Supplementary file 1 — Supplementary Material [file JCMM-26-2766-s001.docx]

**Supplemental materials**


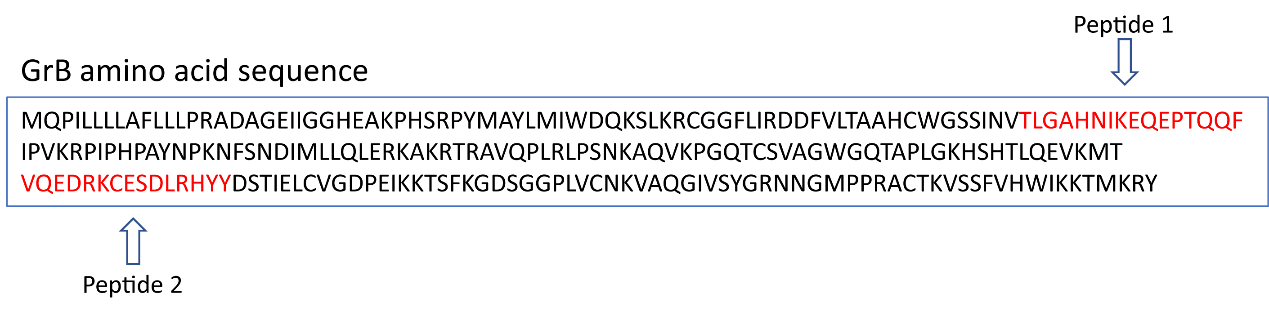


**Figure S1. GrB amino acid sequence**. The sequence was cited from NCBI database (Accession: NM_004131.6). The highlighted sequences are the representative peptides identified by MS in IP products of B cells after the treatment of Figure 5 in the text.


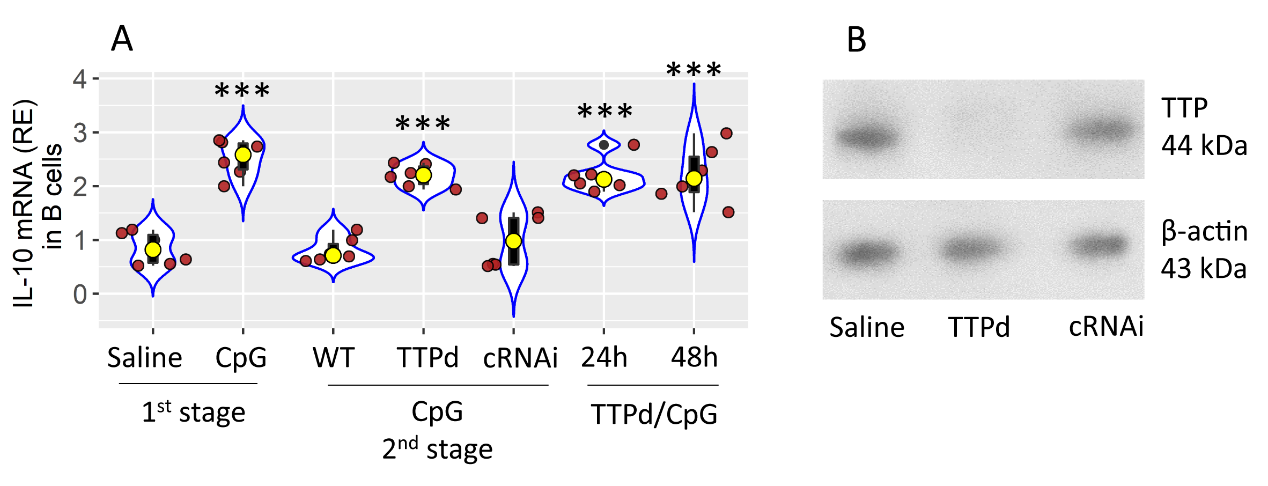


**Figure S2. Inhibition of TTP stabilizes IL-10 mRNA in B cells**. A, naïve CD19^+^ B cells, TTPd (TTP-deficient, made by RNAi) B cells and B cells treated with control RNAi reagents (cRNAi) were cultured in the presence of CpG (1 µg/ml) overnight. Similar to Fig. 3A, the cells were washed and cultured with fresh medium for 4 h, harvested, and the IL-10 mRNA levels in B cells were determined by RT-qPCR. The violin plots show the IL-10 mRNA levels in B cells. The data of violin plots are presented as median (IQR). Each bubble in violin plots presents data obtained from one experiment. ***, p<0.001 (ANOVA + Dunnett’s test), compared with the saline group. B, TTP RNAi results.


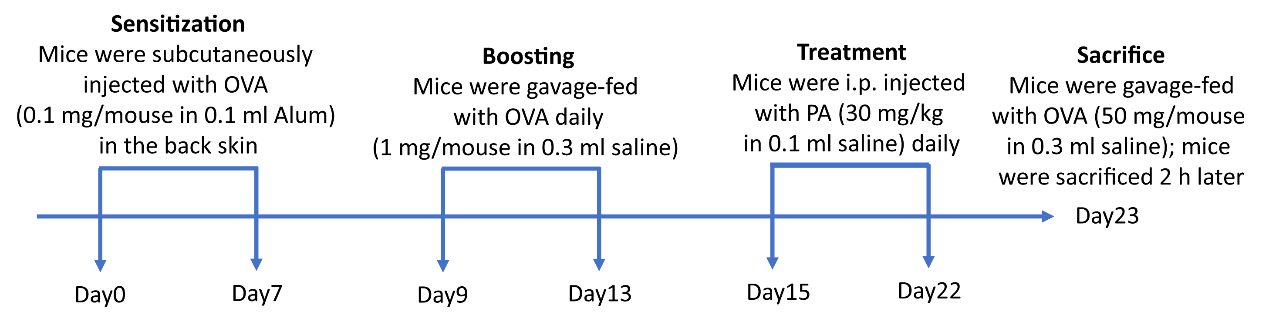


**Figure S3. Procedures of animal model development and treatment with PA**.

**Table S1. Allergens and SPT positive patients**

| **Cow’s milk** | 23 |
| --- | --- |
| **Egg White** | 6 |
| **Egg Yolk** | 2 |
| **Wheat Flour** | 2 |
| **Soy bean** | 6 |
| **Carrot** | 4 |
| **Potato** | 2 |
| **Peanut** | 5 |
| **Almond** | 2 |
| **Cashew** | 2 |
| **Haselnut** | 3 |
| **Sesame** | 5 |
| **Walnut** | 3 |

**Table S2. Serum sIgE levels (U/L)**

| **Patient#** | **sIgE** |  | **Patient#** | **sIgE** |  | **Patient#** | **sIgE** |  | **Patient#** | **sIgE** |
| --- | --- | --- | --- | --- | --- | --- | --- | --- | --- | --- |
| **1** | 53.68 |  | 11 | 92.97 |  | 21 | 62.59 |  | 31 | 90.22 |
| **2** | 49.94 |  | 12 | 101.18 |  | 22 | 93.96 |  | 32 | 76.54 |
| **3** | 96.08 |  | 13 | 62.40 |  | 23 | 81.39 |  | 33 | 70.97 |
| **4** | 37.90 |  | 14 | 90.38 |  | 24 | 68.54 |  | 34 | 75.02 |
| **5** | 89.42 |  | 15 | 37.90 |  | 25 | 58.64 |  | 35 | 109.47 |
| **6** | 101.09 |  | 16 | 58.63 |  | 26 | 68.58 |  | 36 | 45.62 |
| **7** | 41.25 |  | 17 | 59.93 |  | 27 | 66.73 |  | 37 | 87.87 |
| **8** | 79.07 |  | 18 | 65.35 |  | 28 | 45.16 |  | 38 | 66.55 |
| **9** | 55.84 |  | 19 | 68.77 |  | 29 | 49.65 |  | 39 | 46.81 |
| **10** | 75.65 |  | 20 | 48.65 |  | 30 | 34.55 |  | 40 | 65.98 |

**Table S3. SPT wheal size* (mm)**

| **Patient#** |  |  | **Patient#** |  |  | **Patient#** |  |  | **Patient#** |  |
| --- | --- | --- | --- | --- | --- | --- | --- | --- | --- | --- |
| **1** | 9.68 |  | 11 | 6.44 |  | 21 | 14.28 |  | 31 | 14.64 |
| **2** | 7.56 |  | 12 | 12.60 |  | 22 | 11.25 |  | 32 | 7.25 |
| **3** | 11.20 |  | 13 | 11.26 |  | 23 | 12.63 |  | 33 | 11.81 |
| **4** | 8.56 |  | 14 | 15.97 |  | 24 | 11.27 |  | 34 | 7.86 |
| **5** | 11.46 |  | 15 | 9.57 |  | 25 | 7.56 |  | 35 | 9.01 |
| **6** | 13.89 |  | 16 | 8.52 |  | 26 | 12.82 |  | 36 | 15.59 |
| **7** | 8.54 |  | 17 | 10.89 |  | 27 | 10.38 |  | 37 | 11.85 |
| **8** | 12.35 |  | 18 | 12.23 |  | 28 | 6.84 |  | 38 | 5.95 |
| **9** | 5.39 |  | 19 | 11.20 |  | 29 | 5.88 |  | 39 | 14.67 |
| **10** | 9.35 |  | 20 | 8.56 |  | 30 | 6.84 |  | 40 | 10.70 |

*The largest wheal size.
